# Mice, rats, and guinea pigs differ in FMOs expression and tissue concentration of TMAO, a gut bacteria-derived biomarker of cardiovascular and metabolic diseases

**DOI:** 10.1371/journal.pone.0297474

**Published:** 2024-01-24

**Authors:** Klaudia M. Maksymiuk, Mateusz Szudzik, Emilia Samborowska, Dawid Chabowski, Marek Konop, Marcin Ufnal

**Affiliations:** 1 Laboratory of the Centre for Preclinical Research, Department of Experimental Physiology and Pathophysiology, Medical University of Warsaw, Warsaw, Poland; 2 Mass spectrometry Laboratory, Institute of Biochemistry and Biophysics, Polish Academy of Sciences, Warsaw, Poland; Satyawati College, University of Delhi, INDIA

## Abstract

**Introduction:**

Increased plasma trimethylamine oxide (TMAO) is observed in cardiovascular and metabolic diseases, originating from the gut microbiota product, trimethylamine (TMA), via flavin-containing monooxygenases (FMOs)-dependent oxidation. Numerous studies have investigated the association between plasma TMAO and various pathologies, yet limited knowledge exists regarding tissue concentrations of TMAO, TMAO precursors, and interspecies variability.

**Methods:**

Chromatography coupled with mass spectrometry was employed to evaluate tissue concentrations of TMAO and its precursors in adult male mice, rats, and guinea pigs. FMO mRNA and protein levels were assessed through PCR and Western blot, respectively.

**Results:**

Plasma TMAO levels were similar among the studied species. However, significant differences in tissue concentrations of TMAO were observed between mice, rats, and guinea pigs. The rat renal medulla exhibited the highest TMAO concentration, while the lowest was found in the mouse liver. Mice demonstrated significantly higher plasma TMA concentrations compared to rats and guinea pigs, with the highest TMA concentration found in the mouse renal medulla and the lowest in the rat lungs. FMO5 exhibited the highest expression in mouse liver, while FMO3 was highly expressed in rats. Guinea pigs displayed low expression of FMOs in this tissue.

**Conclusion:**

Despite similar plasma TMAO levels, mice, rats, and guinea pigs exhibited significant differences in tissue concentrations of TMA, TMAO, and FMO expression. These interspecies variations should be considered in the design and interpretation of experimental studies. Furthermore, these findings may suggest a diverse importance of the TMAO pathway in the physiology of the evaluated species.

## 1 Introduction

Trimethylamine (TMA) is a gut microbiota metabolite. A plethora of studies show that trimethylamine oxide (TMAO), a product of the liver oxidation of TMA, is a biomarker in cardiovascular [1–3], metabolic [4–6] and renal diseases [7]. TMA is produced by gut microbiota from dietary choline and carnitine [8–13] Another direct source of TMA and TMAO for humans is seafood [14, 15]. TMA crosses the gut-blood barrier and, in the liver, is rapidly oxidized to TMAO by flavin-containing monooxygenase (FMOs). Extensive research has been conducted to elucidate FMOs biochemical properties, substrate specificities, and functional roles. Among them, FMO5 stands out as it exhibits no activity towards trimethylamine [16–23].

In humans, FMO3 in the liver plays a key role in the oxidation of TMA [24]. However, other isoforms of FMO can be found in various organs, such as the kidneys, lungs, heart, and small intestine, in both humans and animals [25–28]. Deficiency in FMO3 activity within the human liver leads to a condition commonly known as "fish odor syndrome" or trimethylaminuria, characterized by symptoms such as fishy body odor [29–32].

The variations in FMO3 activity between mice and rats have been previously reported. Rats exhibit expression of both FMO1 and FMO3 in the liver [27]. In this species, FMO3 is responsible for the oxidation of TMA to TMAO, similar to humans. Conversely, the FMO3 gene expression is suppressed in the liver of male mice, resulting in elevated levels of TMA in their blood and urine [28, 33]. In both male and female mice, FMO1 is postulated to oxidize approximately 10% of TMA [34].

Studies indicate that TMAO could potentially have detrimental effects on various physiological processes and play a direct role in the progression of cardiovascular, renal, and metabolic diseases [2, 5, 35–38]. However, conflicting data from other studies challenge the assertion regarding the negative impact of TMAO [39–41], with some evidence suggesting a positive effect [42–44].

These discrepancies may arise from variations in the doses of TMA/TMAO tested or differences in the physiological levels or metabolism of TMA/TMAO among different species [35–39, 42–50], These factors can lead to differences in TMA/TMAO exposure, potentially influencing the observed effects on health outcomes. Finally, some suggest that TMA but not TMAO exerts a negative effect on the organism [47, 51–55]. Therefore, the expression and activity of FMO3 which oxidizes TMA to TMAO may play a vital role in the biological effects of exogenous TMA and/or TMAO in interventional experimental settings.

Proper selection of animal models is crucial for ensuring translatability of findings to humans, as species-related factors significantly influence data interpretation [56–58].

The interspecies differences in tissue concentrations of TMAO and its precursors in laboratory animals, particularly in rats, mice, and guinea pigs, have not been well-established. This study aimed to determine the tissue concentration of TMAO and its precursors in these commonly used experimental species. Furthermore, the expression of flavin-containing monooxygenases (FMOs) in different tissues was examined to gain insights into TMA/TMAO metabolism in these animals.

## 2 Materials and methods

### 2.1 Animals

The study was performed on 15-17-week-old male *Sprague-Dawley rats*, 10-12-week-old male *BALB/c mice*, and 7-8-month *American guinea pigs*. The age of the animals in our study was selected to reflect their varied lifespans and developmental rates. Rats and mice were obtained from the Central Laboratory for Experimental Animals, Medical University of Warsaw, Poland. Guinea pigs were obtained from Laboratory Animal Breeding of Ilkowice, Poland. The animal study was conducted in the Laboratory of the Centre for Preclinical Research Laboratory, Medical University of Warsaw. The study was performed according to Directive 2010/63 EU on the protection of animals used for scientific purposes and 1st Local Ethics Committee permission no 555/2018 and 464/2017.

The animals were quarantined for 2 weeks after they were brought to the laboratory. The animals were housed in groups of 2–4 in propylene cages, 12 hrs light / 12 hrs dark cycle, temperature 22–23°C, and humidity 45–55%. During this period, the animals were fed a standard laboratory diet for mice, rats (Labofed B standard, Kcynia, Poland) and guinea pigs (Versele-Laga Cavia Complete).

After this period, the animals were anesthetized with urethane (1.5 g/kg BW) during light cycle of the day. Fresh urine samples produced during spontaneous voids before anesthetized were collected. Blood was collected from the heart, and the animals were euthanized by cervical vertebrae dislocation in the case of rats and mice or decapitation in the case of guinea pigs. Feces from the colon were collected and prepared as previously described [59]. The tissue samples: liver, heart, lung, renal cortex, and renal medulla were collected and frozen at -80 degrees Celsius.

### 2.2 Plasma, urine, stools, and tissue TMA and TMAO measurements

The TMA, TMAO, choline and carnitine concentrations in plasma, urine and stools and tissue homogenates were examined using the liquid chromatography-mass spectrometry technique. All urine samples were diluted 10 times using water. Mouse urine samples were further diluted 1000 times or 10 000 times to quantify TMA concentration. Liver, lungs, heart, and kidney (separately the cortex and the medulla) samples were weighed and placed in 10% ethanol (90 μL per 10 mg tissue). Homogenized using the Precellys Cryolys Evolution tissue homogenizer (Bertin Instruments) and stored at -80°C until analysis.

Analyte concentrations were evaluated using Waters Acquity Ultra Performance Liquid Chromatography (Waters, Milford, Massachusetts, USA) coupled with Waters TQ-S triple-quadrupole mass spectrometer (Waters, Manchester, UK). Tissue preparation and the exact determination of metabolites in tissue homogenates have been previously described [52]. The metabolite concentrations in tissues were measured in dry tissue mass.

### 2.3 Real-time PCR

Total RNA was extracted from the lungs, liver, heart, renal cortex and renal medulla using Trizol™ reagent (Thermo Fisher Scientific, Waltham, USA). The RNA concentration and purity (Ratios 260/280 and 260/230) were determined by photometric measurement (NanoPhotometer® N60, Implen, Munich, Germany). The cDNA was produced with an iscript® (Bio-Rad, Hercules, USA) kit according to the manufacturer’s protocol. A Bio-Rad real-time system with iTaq® Universal SYBR Green Supermix (Bio-rad, Hercules, CA, USA) was used to perform real-time PCR using gene-specific primer pairs shown in S1 Table. Primers were designed using the in-silico tool, BLAST. Before the PCR reaction, all primers were analyzed using Gradient PCR to determine the optimum annealing temperature. PCR products were analyzed with a melting curve and agarose gel electrophoresis. The results were calculated by the Delta-Delta Ct method. mRNA Expression levels were normalized relative to the Gapdh- reference gene, selected from four different housekeeping genes using NormFinder software (version 0.953, MOMA, Aarhus, Denmark).

### 2.4 Western blotting

Protein concentration was determined using the Bradford protein assay (Bio-Rad, Hercules, USA). Western blotting was performed in liver, lungs, renal cortex, and renal medulla samples. Protein concentration was determined using the Bradford protein assay (Bio-Rad, Hercules, USA). All samples were resolved using SDS-PAGE gel by electrophoresis. Next proteins were transferred into the PVDF membrane (Bio-Rad, Hercules, USA). The membranes were incubated with skimmed milk for one hour at room temperature, then with different primary antibodies overnight at 4°C, followed by a 1-hour incubation with secondary antibodies labeled with AP. The dilutions of the primary and secondary antibodies are shown in S2 Table. Finally, Quantitative analysis of proteins was performed by ChemiDoc MP Imaging System and Quantity One software (Bio-rad, Hercules, USA).

### 2.5 Statistics

The Shapiro–Wilk test was used to test the normality of the distribution. Differences in the concentrations of choline, carnitine, TMA, and TMAO in the plasma and urine within one species were evaluated by Mann-Whitney U Test. Differences in the concentrations of choline, carnitine, TMA, and TMAO in the tissue homogenates and FMOs expression in the tissues within one species were evaluated by Kruskal-Wallis test, followed by post-hoc Dunn’s test. Differences in the concentrations of choline, carnitine, TMA and TMAO in the plasma, urine and tissue homogenates between the three species were evaluated by Kruskal-Wallis test, followed by post-hoc Dunn’s test. Statistical analysis was conducted using STATISTICA 13.3 (Stat Soft, Krakow, Poland). Outliers were defined (results above Q3 + 1.5 x IQR and below Q1-1.5 x IQR) and removed from the statistical analysis. A value of two-sided p<0.05 was considered significant.

## 3 Results

### 3.1 Part 1—comparison within one species

Plasma and urine concentrations of TMAO and its precursors are presented in Table 1.

**Table 1:** Species-specific comparison of TMAO, TMA and other metabolites concentrations in plasma and urine.

Parameter [μM/L]	Plasma	Urine
**Mice**		
Choline	42.68 (36.54; 48.78)	372.18 (185.54; 464.65)
Carnitine	22.76 (20.65; 26.43)	47.79 (43.38; 146.93)
TMA	1.90 (0.60; 2.35)	17922.01 (345.30; 36429.84)
TMAO	3.68 (3.36; 4.26)	1788.76 (993.74; 3315.89)
**Rats**		
Choline	27.01 (23.39; 40.23)	550.92 (301.78; 778.48)
Carnitine	40.60 (38.37; 43.33)	36.64 (32.26; 49.21)
TMA	<LOQ	3.40 (1.94; 6.19)
TMAO	9.10 (7.41; 9.82)	614.51 (471.73; 798.42)
**Guinea pigs**		
Choline	51.22 (32.06; 67.60)	69.11 (54.19; 97.32)
Carnitine	22.55 (20.03; 32.49)	1.98 (1.29; 2.31)
TMA	0.04 (0.01; 0.07)	33.16 (19.24; 59.62)
TMAO	3.24 (2.73; 9.19)	619.81 (364.75; 1066.12)

Abbreviations: LOQ, Limit of quantification; TMA, trimethylamine; TMAO, trimethylamine oxide. LOQ for TMA in plasma = 0.017 μM/L. All data are expressed as the median.

#### 3.1.1 Mice

The median of TMA and TMAO urine concentrations was 9500-fold and 500-fold higher, respectively, than the plasma concentrations of these substances. The renal cortex and medulla exhibited the highest concentrations of choline and TMA in mouse tissue, respectively. Conversely, the heart and lungs had the lowest concentrations of choline and TMA, respectively. In contrast, the heart had the highest carnitine concentration, while the renal cortex had the lowest concentration. TMAO concentrations in all examined tissues of mice were generally low, with a median below 30 nM/g. The liver had the lowest TMAO concentration, with a median below 3 nM/g (Table 2).

**Table 2:** Species-specific comparison of TMAO and its precursors in the tissue homogenate.

Parameter [nM/g]	Liver	Heart	Lungs	Renal Cortex	Renal medulla	Kruskal-Wallis test
**Mice**						
Choline	3287.15 (2134.77; 4490.67)	409.01 (378.99; 488.68)	1185.42 (1121.19; 1250.79)	5936.21 (5516.04; 6513.84)###	11558.12 (10385.53; 13407.00)###; &&&	P < 0.001
Carnitine	153.86 (129.59; 179.43)	454.72 (429.28; 499.83)*	402.70 (381.97; 416.83)	106.79 (86.63; 129.68)###; &&	121.26 (100.00; 136.62)###; &	P < 0.001
TMA	35.32 (20.13; 47.56)	7.05 (5.79; 9.40)	2.76 (2.30; 2.96)*	48.31 (41.01; 74.24)&&&	70.80 (48.53; 96.49)##, &&&	P < 0.001
TMAO	2.04 (1.69; 5.23)	9.95 (7.86; 48.03)	21.86 (20.61; 33.20)**	20.56 (12.92; 114.54)**	28.75 (11.95; 113.58)**	P = 0.001
**Rats**						
Choline	1818.51 (1417.03; 1893.36)	247.19 (228.96; 262.21)*	867.00 (829.75; 957.90)	2295.91 (1329.02; 2836.27)##	4344.08 (3478.40; 5055.77)###; &&&	P < 0.001
Carnitine	154.24 (132.83; 169.44)	650.07 (638.39; 677.23)	305.01 (291.00; 321.09)	96.46 (91.01; 101.30)###, &&	94.96 (91.31; 99.15)###, &&	P < 0.001
TMA	10.09 (7.97; 12.57)	0.84 (0.75; 1.17)	0.71 (0.60; 0.85)	54.53 (39.55; 86.94)###; &&&	49.56 (44.03; 55.95)##, &&&	P < 0.001
TMAO	26.75 (16.33; 33.03)	3.95 (3.60; 4.28)*	6.66 (5.40; 10.29)	37.90 (21.85; 48.29)##	62.52 (46.84; 118.01)###; &&	P < 0.001
**Guinea pigs**						
Choline	2286.92 (1584.16; 3893.22)	385.60 (259.56; 544.80)	1453.19 (1352.48; 1547.02)	5713.83 (5434.18; 6143.23)###	9505.60 (8140.01; 10647.24)*; ###; &&	P < 0.001
Carnitine	98.79 (91.10; 116.78)	690.72 (639.38; 817.09)***	266.78 (215.78; 285.33)**	149.94 (124.75; 178.84)#	120.96 (104.00; 141.78)###	P < 0.001
TMA	14.02 (4.45; 32.45)	0.99 (0.20; 1.89)**	3.81 (1.57; 4.27)	7.04 (6.94; 8.59)#	12.49 (9.14; 14.06)###; &	P < 0.001
TMAO	9.81 (7.64; 13.98)	3.01 (1.75; 5.64)	6.50 (3.79; 10.96)	16.82 (12.30; 23.46)##	18.15 (11.56; 30.91)##	P < 0.001

Abbreviations: TMA, trimethylamine; TMAO, trimethylamine oxide. All data are expressed as the median, Q1, Q3; Kruskal-Wallis test followed by post-hoc Dunn’s test.

*P < 0.05 vs. liver

**P < 0.01 vs. liver

#P < 0.05 vs. heart

##P < 0.01 vs. heart

###P < 0.001 vs. heart

&P < 0.05 vs. lungs

&&P < 0.01 vs. lungs

&&&P < 0.001 vs. lungs.

**Fig 1:**
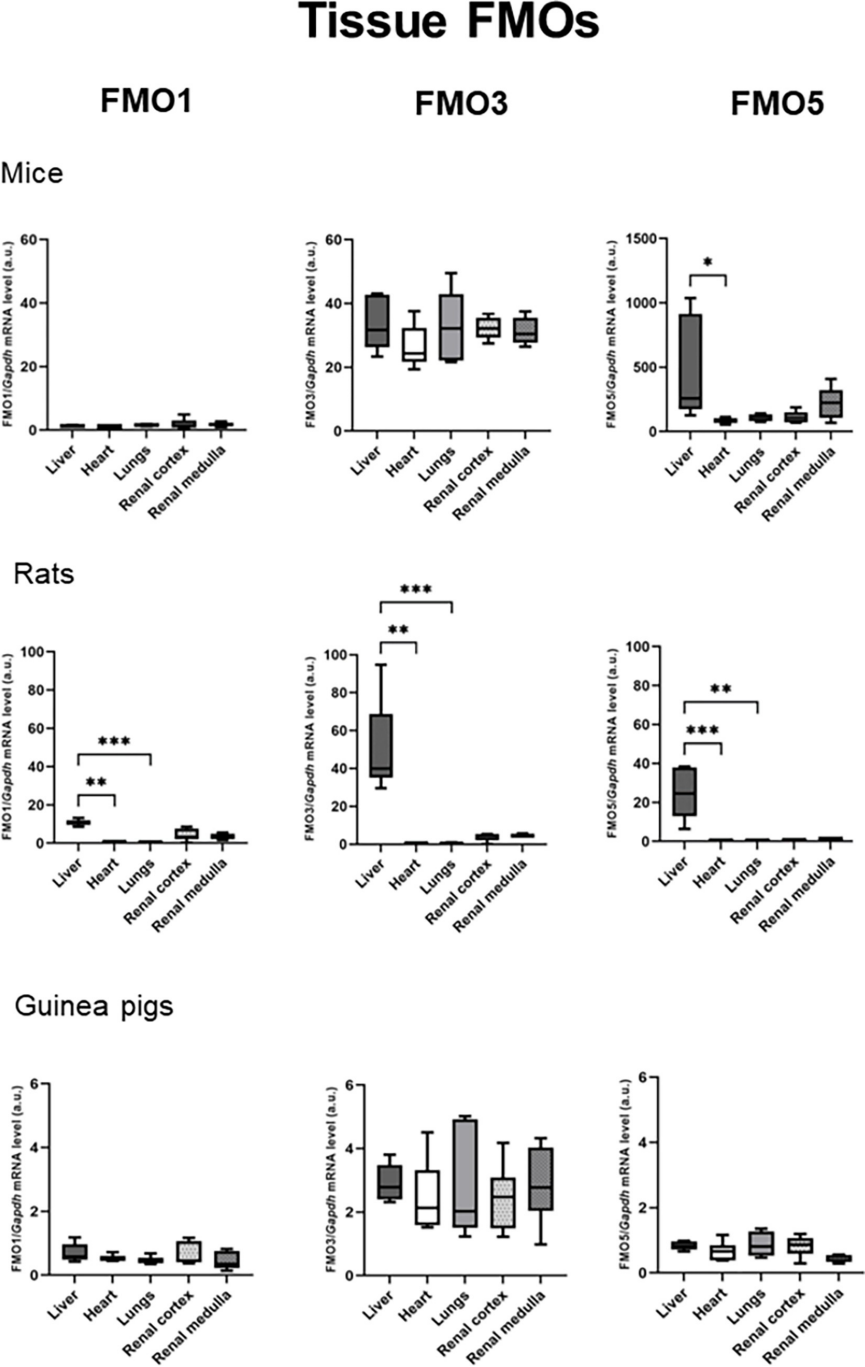
Tissue FMOs. RT-qPCR analysis of FMO1, FMO3 and FMO5 transcript levels in mice, rats and guinea pigs tissues: liver, heart, lungs, renal cortex, renal medulla. Abbreviation: FMO, Flavin-containing monooxygenase. All data are expressed as the median, Q1, Q3, MIN, MAX (n = 5 or 6; use arbitrary units); Kruskal-Wallis test followed by post-hoc Dunn’s test; *P<0.05; **P<0.01; ***P<0.001.

Among the examined tissues, mouse liver displayed the highest expression of the FMO5 gene. Additionally, mice exhibited FMO3 gene expression in all the examined tissues, albeit at lower levels compared to FMO5 gene expression (Fig 1).

#### 3.1.2 Rats

The choline concentration in urine was found to be 20-fold higher than in plasma, while the TMAO concentration in urine was 67-fold higher than in plasma. Plasma TMA levels were below the limit of quantification, indicating very low levels of TMA in the blood (Table 1). The renal cortex and medulla exhibited the highest concentrations of choline, TMA, and TMAO, while the heart and lungs had the lowest concentrations of these substances. In contrast, the heart had the highest carnitine concentration, whereas the renal cortex and medulla had the lowest concentrations (Table 2).

Among the FMO genes, FMO1, FMO3, and FMO5 showed the highest expression levels in the livers of rats. Notably, the FMO3 gene exhibited the highest expression among all the FMO genes. Minimal expression of FMO1 and FMO3 was observed in the renal cortex and medulla of rats (Fig 1).

#### 3.1.3 Guinea pigs

In guinea pigs, the plasma carnitine concentration was found to be 11-fold higher than in urine. Conversely, the median concentrations of TMA and TMAO were significantly higher in urine compared to plasma, with increases of over 800-fold and almost 200-fold, respectively (Table 1). The renal medulla exhibited the highest choline concentration, while the heart had the lowest concentration. The heart had the highest carnitine concentration, whereas the liver had the lowest concentration. Guinea pigs displayed low concentrations of TMA and TMAO in all examined tissues, with median concentrations below 20 nM/g. The liver had the highest TMA concentration, while the renal medulla had the highest TMAO concentration. The heart had the lowest concentrations of both TMA and TMAO (Table 2).

Guinea pigs exhibited low expression of the FMO genes in the examined tissues, with no significant difference observed between the tissues. FMO3 showed the highest expression among the FMO genes (Fig 1).

### 3.2 Part 2 –Interspecies comparison

#### 3.2.1 TMA and TMAO concentrations in plasma and tissues

**Fig 2:**
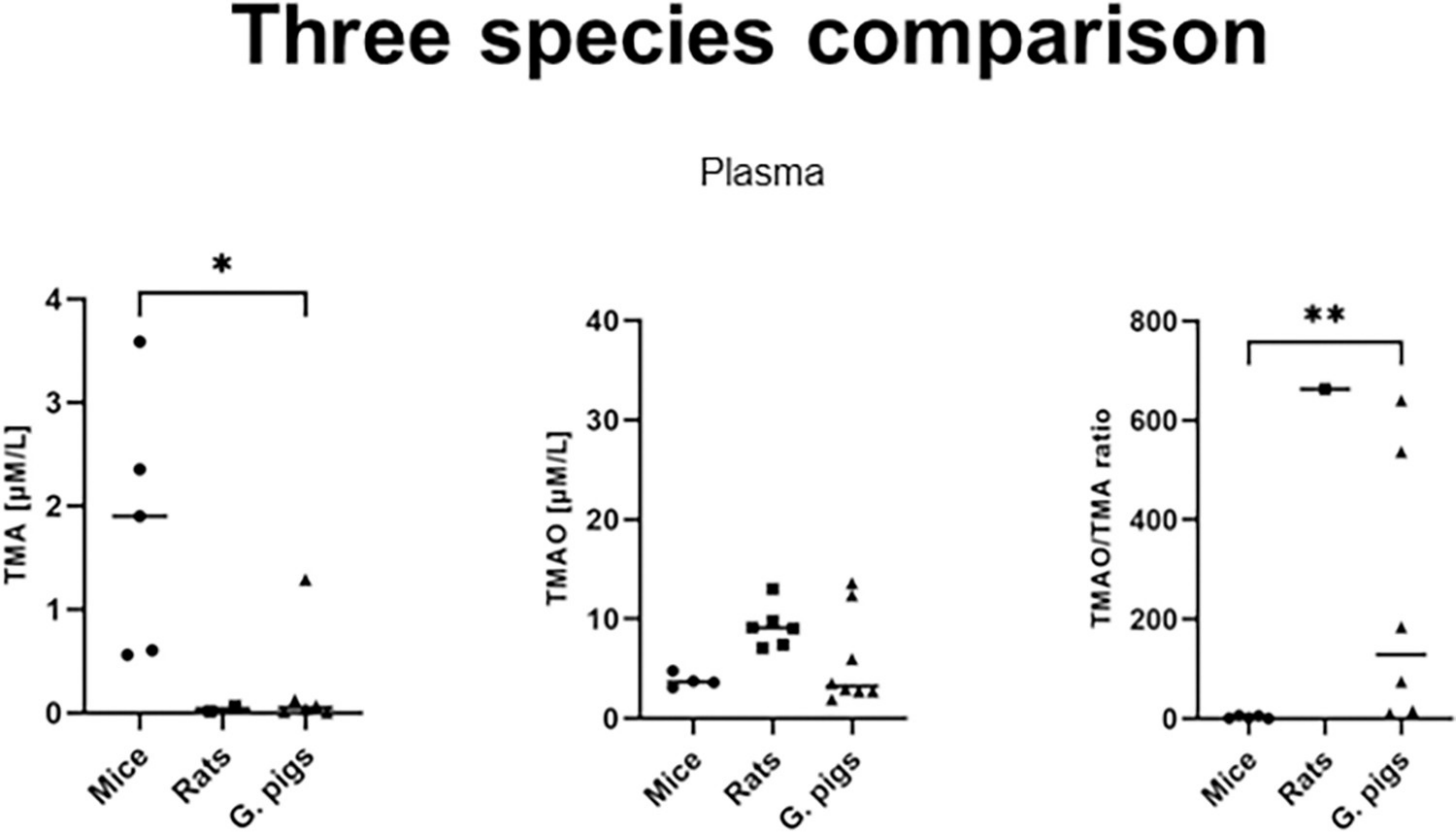
Three species comparison—Plasma. Comparison of the concentrations of TMA, TMAO and the TMAO/TMA ratio in the plasma. Abbreviations: TMA, Trimethylamine; TMAO, Trimethylamine oxide. LOQ for TMA in plasma = 0.017 μM/l. All data are expressed as the individual values and median; Kruskal-Wallis test followed by post-hoc Dunn’s test; *P<0.05; **P<0.01.

Rats exhibited a higher plasma concentration of creatinine compared to mice and guinea pigs (Table 3). Mice displayed significantly higher plasma TMA concentration than guinea pigs, while also showing a significantly lower TMAO/TMA ratio in plasma. Plasma TMA levels in rats were below the limit of quantification. All study groups demonstrated a similar median plasma concentration of TMAO (Fig 2).

**Table 3:** Species-specific comparison of TMAO, TMA, and other metabolites plasma and urine concentrations.

Parameter [μM/L]	Mice	Rats	Guinea pigs	Kruskal- Wallis test
**Plasma**				
Choline	42.68 (36.54; 48.78)	27.01 (23.39; 40.23)	51.22 (32.06; 67.60)	P = 0.09
Carnitine	22.76 (20.65; 26.43)	40.60 (38.37; 43.34)*	22.55 (20.03; 32.49)#	P = 0.007
**Urine**				
Choline	372.18 (185.54; 464.65)	550.92 (301.78; 778.49)	69.11 (54.19; 97.32)*, ###	P < 0.001
Carnitine	47.79 (43.38; 146.93)	36.64 (32.26; 49.21)	1.98 (1.29; 2.31)***, #	P < 0.001
TMA	17922.01 (345.30; 36429.84)	3.40 (1.94; 6.19)***	33.16 (19.24; 59.62)	P < 0.001
TMAO	1788.76 (993.74; 3315.89)	614.51 (471.73; 798.42)*	619.81 (364.75; 1066.12)*	P = 0.009
TMAO/TMA ratio	0.10 (0.09; 3.60)	225.51 (58.47; 282.12)***	13.34 (10.58; 26.75)	P < 0.001
**`Stools**				
Choline	72.71 (60.99; 130.64)	19.90 (15.86; 55.94)*	60.84 (34.14; 127.10)	P = 0.02
Carnitine	0.85 (0.52; 1.40)	0.28 (0.17; 1.19)	0.57 (0.38; 0.64)	P = 0.18
TMA	51.16 (32.06; 59.87)	14.41 (9.18; 16.87)*	5.96 (1.75; 10.50)**	P = 0.001
TMAO	<LOQ	<LOQ	<LOQ	-

All data are expressed as the median, Q1, Q3; Kruskal-Wallis test followed by post-hoc Dunn’s test.

*P < 0.05 vs. mice

**P < 0.01 vs. mice

***P < 0.001 vs. mice

#P < 0.05 vs. rats

###P < 0.001 vs. rats.

**Fig 3:**
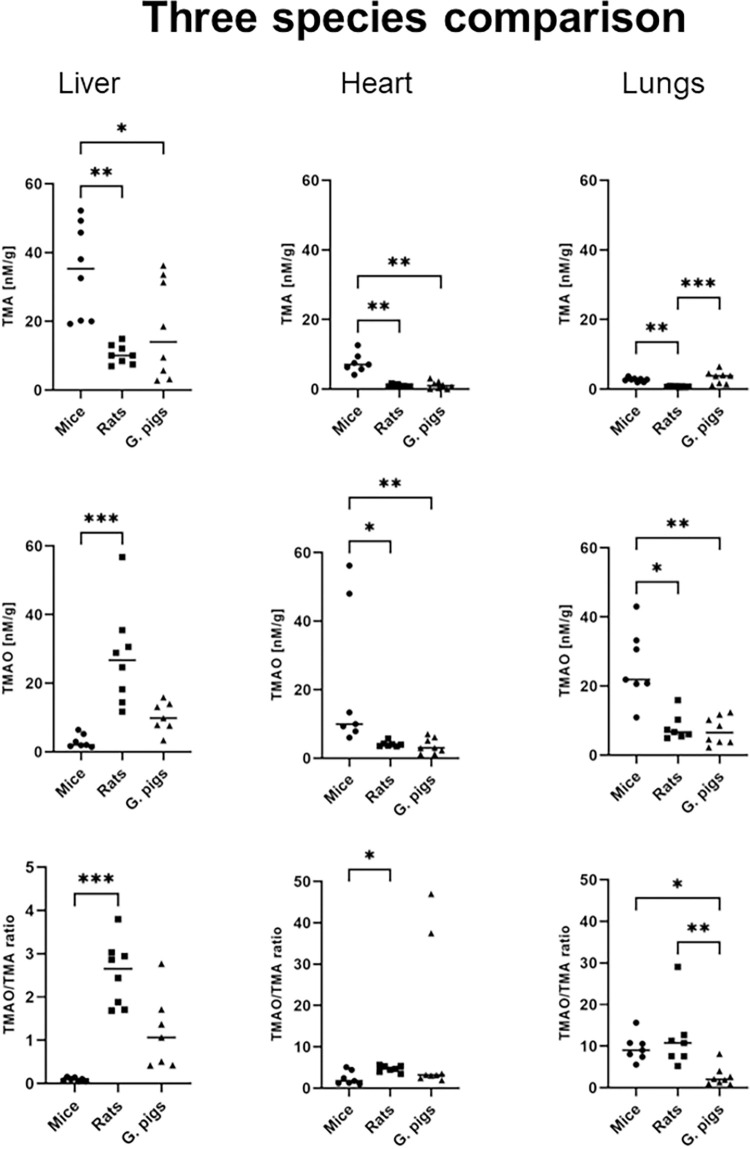
Three species comparison—Tissues. Comparison of the concentrations of TMA, TMAO and the TMAO/TMA ratios in the liver, heart and lungs. Abbreviations: TMA, Trimethylamine; TMAO, Trimethylamine oxide. All data are expressed as the individual values and median; Kruskal-Wallis test followed by post-hoc Dunn’s test; *P<0.05; **P<0.01; ***P<0.001.

**Fig 4:**
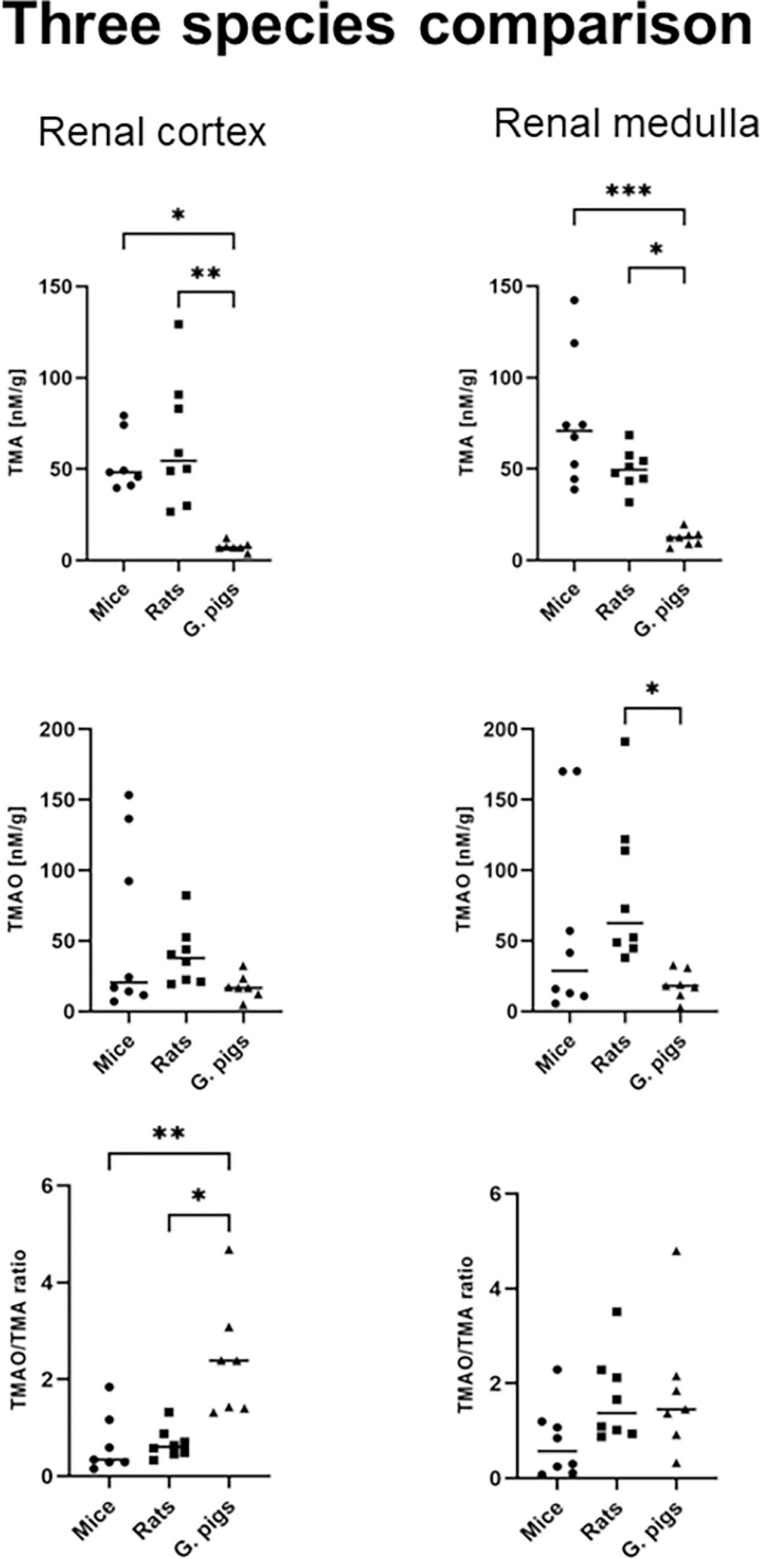
Three species comparison–Tissues, continuation. Comparison of the concentrations of TMA, TMAO and the TMAO/TMA ratios in the renal cortex and renal medulla. Abbreviations: TMA, Trimethylamine; TMAO, Trimethylamine oxide. All data are expressed as the individual values and median; Kruskal-Wallis test followed by post-hoc Dunn’s test; *P<0.05; **P<0.01; ***P<0.001.

In terms of TMA concentration in the liver, mice exhibited higher levels compared to rats and guinea pigs, with a 3.5-fold and 2.5-fold difference, respectively. However, the TMAO concentration in the liver of mice was 13-fold lower than that of rats. The TMAO/TMA ratio in the liver was almost 30-fold higher in rats compared to mice (Fig 3). Mice displayed higher TMA concentration in the heart compared to rats and guinea pigs, with an 8-fold and 7-fold difference, respectively. The TMAO concentration in the heart was 3-fold higher in mice compared to rats and guinea pigs. Additionally, the TMAO/TMA ratio in the heart was significantly higher in rats compared to mice (Fig 3). Rats exhibited lower TMA concentration in the lungs compared to mice and guinea pigs, with a 4-fold and 5-fold difference, respectively. The median TMAO concentration in the lungs was 3-fold higher in mice compared to rats and guinea pigs. Furthermore, guinea pigs had a 5-fold lower TMAO/TMA ratio in the lungs compared to mice and rats (Fig 3). In the renal cortex, guinea pigs displayed lower TMA concentration compared to mice and rats, with a 7-fold and 8-fold difference, respectively. The TMAO/TMA ratio in the renal cortex was higher in guinea pigs compared to mice and rats, with a 7-fold and 4-fold difference, respectively. There were no significant differences observed between the study groups in terms of TMAO concentration in the renal cortex (Fig 4). The median TMA concentration in the renal medulla was lower in guinea pigs compared to mice and rats, with a 6-fold and 4-fold difference, respectively. The TMAO concentration in the renal medulla was 3-fold higher in rats compared to guinea pigs. No significant differences were found between the groups in terms of the TMAO/TMA ratio in the renal medulla (Fig 4).

#### 3.2.2 Protein quantification of FMO3 and FMO5

**Fig 5:**
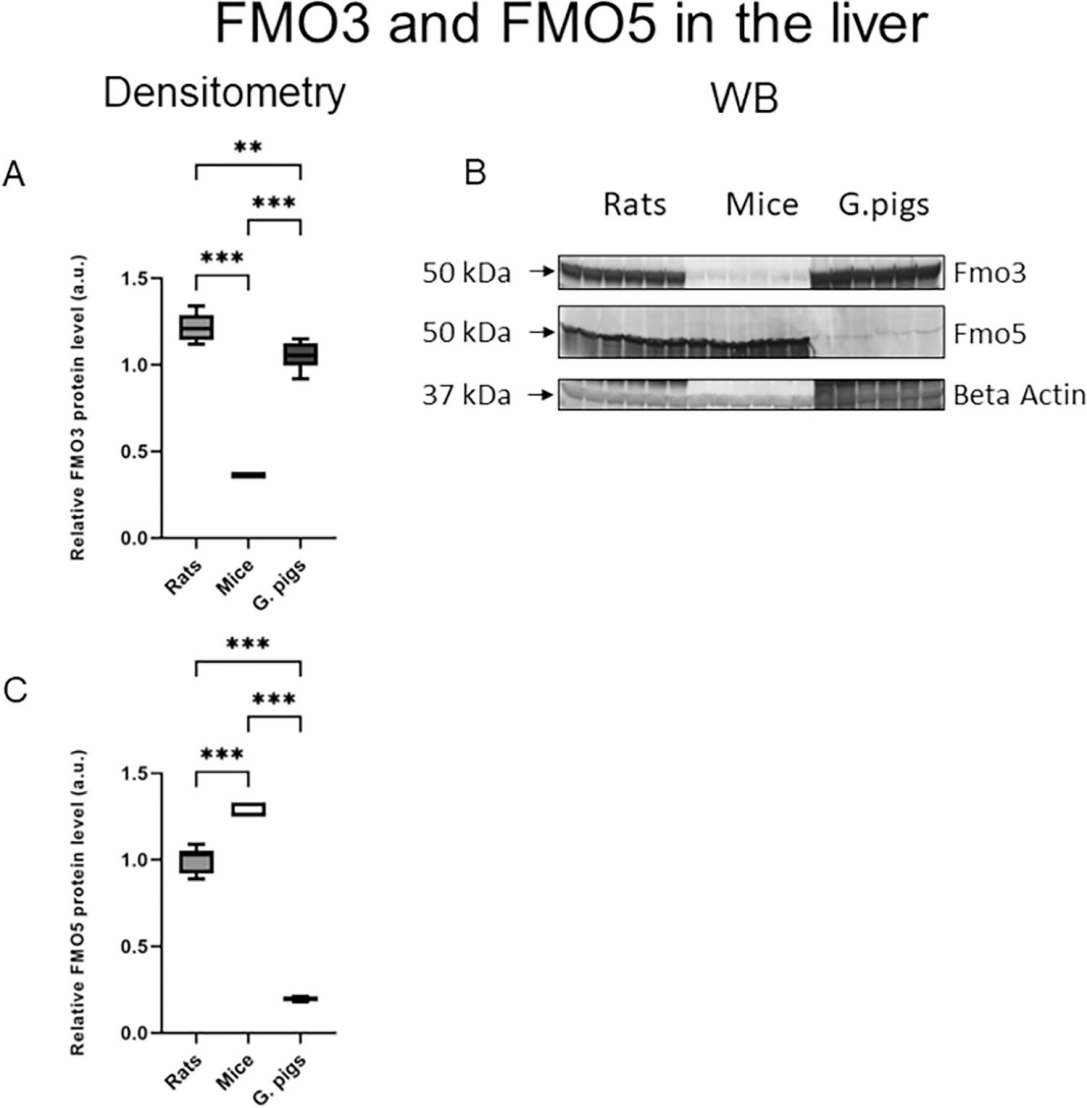
FMO3 and FMO5 in the liver. Western blot analysis of FMO3 (A) and FMO5 (C) protein levels from total protein extract prepared from the liver in rats, mice and guinea pigs. (B) A representative immunoblot. Immunolabeled FMO3 and beta actin loading control bands were quantified using a Molecular Imager. Relative levels of the test proteins are plotted in arbitrary units. Abbreviations: FMO, Flavin-containing monooxygenase. All data are expressed as the median, mean, Q1, Q3, MIN, MAX (n = 4–6); ANOVA followed by post-hoc Tuckey test. **P < 0.01; ***P < 0.001.

**Fig 6:**
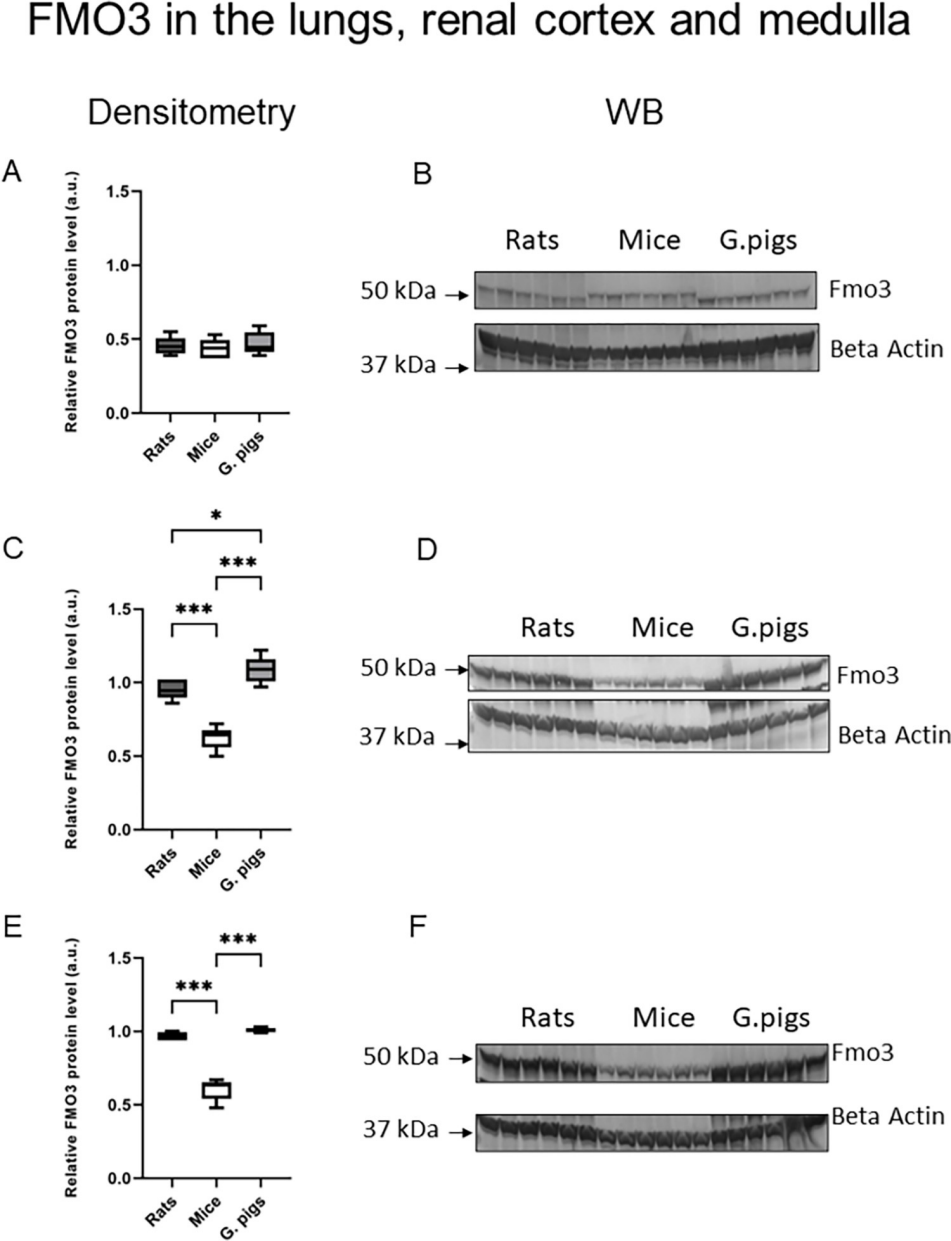
FMO3 in the lungs, renal cortex and medulla. Western blot analysis of FMO3 protein level from total protein extract prepared and a representative immunoblot from the (A, B) lungs (C. D) renal cortex and (E, F) renal medulla in rats, mice and guinea pigs. Immunolabeled FMO3 and beta actin loading control bands were quantified using a Molecular Imager. Relative levels of the test proteins are plotted in arbitrary units. Abbreviations: FMO, Flavin-containing monooxygenase. All data are expressed as the median, mean, Q1, Q3, MIN, MAX (n = 5–6); ANOVA followed by post-hoc Tuckey test. *P < 0.05; ***P < 0.001.

The levels of FMO3 protein in the liver (Fig 5), renal cortex, and renal medulla (Fig 6) were significantly higher in rats and guinea pigs compared to mice. Furthermore, rats exhibited a significantly higher level of FMO3 protein in the liver and a significantly lower level in the renal cortex compared to guinea pigs. On the other hand, the level of FMO5 protein in the liver (Fig 5) was highest in mice. Rats also showed a high level of FMO5 protein in the liver.

#### 3.2.3 Choline and carnitine concentrations in plasma and tissues

Among the examined tissues, rats exhibited the lowest choline concentrations compared to mice or guinea pigs. Mice had the lowest concentration of carnitine in the heart and the highest concentration in the lungs. Rats displayed the lowest carnitine concentration in the renal medulla. Guinea pigs had the lowest concentration of carnitine in the liver and the highest concentration in the renal cortex (Table 4).

**Table 4:** Species-specific comparison of choline and carnitine concentrations in various tissues.

Parameter [μM/kg]	Mice	Rats	Guinea pigs	Kruskal-Wallis test
**Liver**				
Choline	3287.15 (2134.77; 4490.67)	1818.51 (1417.03; 1893.36)*	2286.92 (1584.16; 3893.22)	P = 0.02
Carnitine	153.86 (129.59; 179.43)	154.24 (132.83; 169.44)	98.79 (91.10; 116.78)**, #	p = 0.002
**Heart**				
Choline	409.01 (378.99; 488.68)	247.19 (228.96; 262.21)*	385.60 (259.56; 544.80)	P = 0.01
Carnitine	454.72 (429.28; 499.83)	650.07 (638.39; 677.23)*	690.72 (639.38; 817.09)**	P = 0.002
**Lungs**				
Choline	1185.42 (1121.19; 1250.79)	867.00 (829.75; 957.90)	1453.19 (1352.48; 1547.02)###	P < 0.001
Carnitine	402.70 (381.97; 416.83)	305.01 (291.00; 321.09)*	266.78 (215.78; 285.33)***	P < 0.001
**Renal cortex**				
Choline	5936.21 (5516.04; 6513.84)	2295.91 (1329.02; 2836.27)**	5713.83 (5434.18; 6143.23)##	P < 0.001
Carnitine	106.79 (86.63; 129.68)	96.46 (91.01; 101.30)	149.94 (124.75; 178.84)*, ##	P = 0.003
**Renal medulla**				
Choline	11558.12 (10385.53; 13407.00)	4344.08 (3478.40; 5055.77)***	9505.60 (8140.01; 10647.24)#	P < 0.001
Carnitine	121.26 (100.00 136.62)	94.96 (91.31; 99.15)*	120.96 (104.00; 141.78)	P = 0.02

All data are expressed as the median, Q1, Q3; Kruskal-Wallis test followed by post-hoc Dunn’s test.

*P < 0.05 vs. mice

**P < 0.01 vs. mice

***P < 0.001 vs. mice

#P < 0.05 vs. rats

##P < 0.01 vs. rats

###P < 0.001 vs. rats.

## 4 Discussion

The key finding of our study is the significant variation in tissue concentration of TMA and TMAO among mice, rats, and guinea pigs, despite comparable plasma levels of TMAO. Additionally, we observed pronounced differences in the concentrations of methylamines across different organs and tissues. Specifically, the kidneys displayed the highest concentrations, while the lungs and hearts exhibited the lowest concentrations of these compounds.

TMAO originates mostly from TMA, a gut microbiota product of dietary choline and carnitine [8–13]. TMA crosses the gut-blood barrier and is oxidized to TMAO by hepatic FMO3 [24]. The expression of the gene encoding FMO3, is switched off in the liver of male mice at the age of 5–6 weeks [28, 33]. In male mice, hepatic FMO1 is responsible for approximately 10% of TMA oxidation to TMAO. These factors contribute to the higher concentration of TMA in mice plasma compared to other species [34].

Increasing evidence suggests that plasma TMAO is a marker of cardiovascular, renal, and metabolic diseases [1–7, 60]. Furthermore, findings from multiple studies suggest a potential involvement of TMAO and TMA in the development of the aforementioned diseases. However, it is crucial to acknowledge the current lack of clinical interventional studies in humans. The existing experimental interventional studies, conducted in diverse animal species and under varying experimental conditions, have produced conflicting results and interpretations [39–44].

The present study aimed to address the current gap in knowledge regarding inter-species differences in tissue concentrations of TMAO, TMA, and their precursors in laboratory animals. Here, we provide comprehensive data on the levels of carnitine, choline, TMA, and TMAO in different tissues and body fluids harvested from the three commonly used species of laboratory animals. Importantly, this study represents the first attempt to evaluate and compare the concentrations of these metabolites across various tissues and body fluids in these three species, which are most commonly utilized in the generation of animal models for disease in pre-clinical studies.

Choline and carnitine in mammals are obtained from the diet and, to a lesser extent, through endogenous synthesis [61, 62]. Intestinal bacteria metabolize dietary choline and carnitine that are not absorbed in the small intestine, leading to the formation of TMA. In the current study, rats exhibited significantly lower choline concentrations in tissues compared to the other two species. However, plasma choline levels were similar among the three species. Notably, choline concentration in urine was consistently higher than in plasma in all tested species. Additionally, the kidneys showed the highest choline concentration, suggesting that the kidneys play a primary role in choline excretion [63]. In all three species, the heart exhibited the highest concentration of carnitine, potentially highlighting its primary role in transporting long-chain fatty acids to the mitochondrial matrix. This process is essential for beta-oxidation and the subsequent production of energy from fatty acids [64]. Interestingly, we found that guinea pigs had a tenfold higher concentration of carnitine in plasma compared to urine. This finding suggests that carnitine is a highly conserved compound in guinea pigs, with a significant portion being retained rather than excreted in the urine.

### 4.1 TMA and TMAO concentration

Tissue and plasma levels of TMA and TMAO are influenced by the absorption of TMA, its subsequent oxidation to TMAO, and the excretion of both amines. In the present study, mice consistently exhibited the highest TMA levels in their tissues. Moreover, mice demonstrated the highest concentration of TMA in their stool. Based on these findings, we speculate that the elevated microbial production of TMA in the gut could be an additional factor contributing to the high plasma concentrations of this compound in mice. To further elucidate the role of gut microbiota in TMA production, examining TMA concentrations in the blood from the portal vein across these species would be informative.

The highest concentrations of TMA among the examined tissues were consistently observed in the renal cortex and medulla, supporting the notion that these organs serve as major routes for the excretion of TMA and TMAO [65–67].

In our study, the plasma concentration of TMAO in mice, rats, and guinea pigs ranged between 5 and 10 μM/L (Table 1), which is in line with previously reported levels in healthy individuals and animals [2, 3, 35, 36, 38, 42, 68–73]. Notably, the plasma concentration of TMAO was comparable across all three species, despite significant differences in tissue concentrations of TMA and TMAO. Variations in the rates of TMA to TMAO oxidation, tissue clearance, and urine excretion of these compounds may contribute to this phenomenon. The urinary excretion of TMA and TMAO plays a crucial role in maintaining low plasma levels of these metabolites. Numerous studies have consistently demonstrated that impaired kidney function is associated with elevated plasma levels of TMA and TMAO [7, 65, 70, 74]. Therefore, given the significant differences in tissue concentrations of TMA and TMAO among the three species, but similar plasma levels of these methylamines, it appears that plasma TMAO level could potentially serve as a biomarker for kidney function, akin to plasma creatinine or cystatin [75–78]. On the other hand, tissue concentrations of TMA and TMAO may provide more valuable insights into their potential biological roles and functions within the body, stressing also interspecies differences.

### 4.2 FMO expression and TMA/TMAO oxidation

In humans, it is widely recognized that FMO3 in the liver plays a significant role in the oxidation of TMA to TMAO [24]. Our study shows that rats have the highest level of FMO3 protein and the highest TMAO/TMA ratio in the liver, compared to mice and guinea pigs. Moreover, rats exhibited FMO5 gene expression and a high level of FMO5 protein in the liver. Notably, we also detected FMO1 and FMO3 gene expression in the renal cortex and renal medulla of rats, along with the highest TMAO/TMA ratio in the renal medulla. These findings suggest that TMA is metabolized to TMAO in the kidneys of rats as well. Other studies have reported FMO1 expression in the kidneys of both humans and rats [79, 80]. The TMAO/TMA ratio in urine was highest in rats. However, both rats and guinea pigs exhibited ratios above 1, confirming effective TMA metabolism to TMAO in these species.

Guinea pigs exhibited lower levels of FMO3 protein and a lower TMAO/TMA ratio in the liver compared to rats. Additionally, they had the lowest level of FMO5 protein in the liver among all the species studied. However, guinea pigs demonstrated the highest level of FMO3 protein in the renal cortex and renal medulla compared to the other two species. Moreover, the TMAO/TMA ratios were above 1 in both the renal cortex and renal medulla of guinea pigs. These findings suggest that kidney oxidation likely plays an important role in the metabolism of TMA to TMAO in guinea pigs.

We investigated the expression of FMO1, FMO3, and FMO5 in five different tissues. Among the studied species, mice exhibited the highest expression of FMO5 in the liver. Conversely, mice demonstrated low expression levels of FMO1 and FMO3 in the liver as well as other tissues. Furthermore, mice had the lowest level of FMO3 protein and the highest level of FMO5 protein in the liver compared to the other species examined. These findings support the notion that in the liver of male mice, FMO3 is not actively involved in TMA metabolism [28, 33]. Janmohamed et al. showed that the most expressed FMO in the mice liver is FMO5, which is consistent with our results [28]. Further studies investigating the role of FMO5 in male mice are warranted to gain a deeper understanding of its function in TMA metabolism. Additionally, it is noteworthy that in our study in most tissues of mice, the TMAO/TMA ratio was below 1. This observation suggests that TMA metabolism is relatively low in the overall mouse body.

Several studies suggest that the guinea pig may be the preferred model for preclinical cardiovascular research due to its lipoprotein profile, cholesterol metabolism, and heart electrophysiology, which closely resemble those of humans [81–84]. To date, experimental studies investigating the role of TMAO in cardiovascular disease in guinea pigs are lacking. This study establishes the plasma and tissue concentration of TMAO and its precursors in guinea pigs for the first time, paving the way for further research on evaluating the impact of TMAO in cardiovascular and other diseases using the guinea pig model.

The limitation of our study lies in the absence of calculations for the total 24-hour turnover of TMAO and its precursors. Such calculations would necessitate precise measurements of 24-hour intake and excretion in stools, urine, and exhaled air. A more com-prehensive understanding of TMAO metabolism would involve evaluating the metabolism of TMAO precursors in the intestinal tract, the efficacy of the gut-blood barrier, and metabolism within the liver, kidney, and other tissues.

In this study, we analyzed the concentrations of TMAO and its precursors across various tissues in rats, mice, and guinea pigs. While plasma TMAO levels were similar, we noted marked differences in tissue concentrations of TMA, TMAO, and FMOs expression among the three species. This underscores the interspecies variations in the TMAO pathway, suggesting possible differences in the biological responses to either activation or inhibition of this pathway through experimental procedures. Recognizing the critical role of species selection in pre-clinical study design for effective translational research [56, 57], our findings provide valuable insights to guide the choice of animal species in upcoming studies.

## 5 Conclusion

In conclusion, our research provides new data on the distinct concentrations of TMA precursors, TMA, and TMAO in plasma, urine, and tissues across mice, rats, and guinea pigs. Uniquely, we found that despite similar plasma TMAO levels, tissue concentrations of TMA and TMAO significantly varied across these species, indicating crucial interspecies differences. This variation in tissue methylamine concentrations suggests potential disparities in the physiological importance and roles of these compounds, as well as possible differences in biological responses to exogenous TMA and TMAO. Such understanding could have notable implications for future research directions, especially in studies focusing on the role of TMA and TMAO in cardiovascular, renal, and metabolic diseases. Future preclinical studies should take into account interspecies differences, particularly in the context of FMOs expression and TMA and TMAO levels. This approach would help ensure that the research outcomes are more representative and reflective of the biological diversity and would enable a more comprehensive understanding of the precise functions of TMA and TMAO in disease pathogenesis.

## Supporting information

S1 Table List of oligonucleotide primers used for RT-qPCR. (DOCX)

S2 Table List of antibodies used for western blot analyses. (DOCX)

S3 Table Raw data. (PDF)

S1 Fig Western blotting–original images. (PDF)
