# Supplementary material for: Mice, rats, and guinea pigs differ in FMOs expression and tissue concentration of TMAO, a gut bacteria-derived biomarker of cardiovascular and metabolic diseases
Source: PLoS One. 2024 Jan 24;19(1):e0297474. doi: 10.1371/journal.pone.0297474 (PMC10807837; doi:10.1371/journal.pone.0297474)
Supplement: S1 Table — (DOCX) [file pone.0297474.s001.docx]

**S1 Table.** List of oligonucleotide primers used for RT-qPCR

| **RAT** | | | | |
| --- | --- | --- | --- | --- |
| **Gene** | **Encoded product** | **Unique Biorad Assay ID** | **PCR product size [bp]** | **Accession**  **number** |
| *Fmo1* | flavin containing dimethylaniline monoxygenase 1 | qRnoCID0008990 | 103 | ENSRNOG00000034191 |
| *Fmo3* | flavin containing dimethylaniline monoxygenase 3 | qRnoCID0003196 | 92 | ENSRNOG00000003620 |
| *Fmo5* | flavin containing dimethylaniline monoxygenase 5 | qRnoCID0053250 | 114 | ENSRNOG00000018076 |
| *Gapdh* | glyceraldehyde-3-phosphate dehydrogenase | qRnoCID0057018 | 115 | ENSRNOG00000018630 |
| **MOUSE** | | | | |
| **Gene** | **Encoded product** | **Primers** | **PCR product size [bp]** | **Accession**  **number** |
| *Fmo1* | flavin containing dimethylaniline monoxygenase 1 | F:AGACCACAAGTGAGTGAACG  R:CACTTCCCACTGTCCAGAGAC | 431 | XM_006496663.4 |
| *Fmo3* | flavin containing dimethylaniline monoxygenase 3 | F:AACAACTTACCCACCGCCAT  R:TGAACACGGGCTCTTTCCTG | 121 | NM_008030.2 |
| *Fmo5* | flavin containing dimethylaniline monoxygenase 5 | F:TGTGGCCTGATGTGTGTTCA  R:AAATACTGGCCCTGCCTTCC | 253 | NM_001161765.1 |
| *Gapdh* | glyceraldehyde-3-phosphate dehydrogenase | F:CCCTTAAGAGGGATGCTGCC  R:ACTGTGCCGTTGAATTTGCC | 263 | NM_001289726.1 |
| **GUINEA PIG** | | | | |
| **Gene** | **Encoded product** | **Primers** | **PCR product size [bp]** | **Accession**  **number** |
| *Fmo1* | flavin containing dimethylaniline monoxygenase 1 | F:AAGGGTGGAACCACATCACC  R:GCAACTCGTTTGGCCATGTT | 173 | XM_003474740.4 |
| *Fmo3* | flavin containing dimethylaniline monoxygenase 3 | F:GAAAACGCAGCCAGGAGCTA  R:CTGATGGAGGCCAAACCACT | 238 | XM_013158075.2 |
| *Fmo5* | flavin containing dimethylaniline monoxygenase 5 | F:TGCTGCTGATGATGATGGTAGT  R:TGGGATTGGGTAGTCGCTGA | 441 | NM_001172947.1 |
| *Gapdh* | glyceraldehyde-3-phosphate dehydrogenase | F:TGGTGAAGGTCGGAGTGAAC  R:TCCCATTCTCAGCCTTGACG | 189 | NM_001172951.1 |
