# Supplementary material for: Mice, rats, and guinea pigs differ in FMOs expression and tissue concentration of TMAO, a gut bacteria-derived biomarker of cardiovascular and metabolic diseases
Source: PLoS One. 2024 Jan 24;19(1):e0297474. doi: 10.1371/journal.pone.0297474 (PMC10807837; doi:10.1371/journal.pone.0297474)
Supplement: S2 Table — (DOCX) [file pone.0297474.s002.docx]

**S2 Table.** List of antibodies used for Western blot analyses.

| **Target**  **protein** | **Primary Ab** | **Dilution** | **Secondary Ab** | **Dilution** |
| --- | --- | --- | --- | --- |
| Fmo3 | Rabbit polyclonal, Abcam  ab126711 | 1:2000 | Goat anti-rabbit, Abcam  ab97048-1 | 1:10,000 |
| Fmo5 | Rabbit polyclonal, Invitrogen  PA5-79277 | 1:2000 | Goat anti-rabbit, Abcam  ab97048-1 | 1:10,000 |
| Beta-Actin | Goat polyclonal, Abcam  ab8229 | 1:2000 | Donkey anti-goat, Abcam  ab 97107 | 1:10,000 |
