# Supplementary material for: Mice, rats, and guinea pigs differ in FMOs expression and tissue concentration of TMAO, a gut bacteria-derived biomarker of cardiovascular and metabolic diseases
Source: PLoS One. 2024 Jan 24;19(1):e0297474. doi: 10.1371/journal.pone.0297474 (PMC10807837; doi:10.1371/journal.pone.0297474)
Supplement: S3 Table — (PDF) [file pone.0297474.s003.pdf]

S3 Table. Raw data.

Group	ID	TMA - plasma [uM/l]	TMAO-plasma [uM/l]	choline - plasma [uM/l]	carnitine - plasma [uM/l]	TMA - urine [uM/l]	TMAO-urine [uM/l]	choline - urine [uM/l]	carnitine - urine [uM/l]	TMA - stool [uM/l]	choline - stool [uM/l]	carnitine - stool [uM/l]
rats	K21		7.41	40.23	36.14	3.40	958.27	773.16	31.03			
rats	K22		9.15	27.01	38.99	1.24	583.87	301.92	33.50	11.45	19.90	0.21
rats	K23		13.02	43.76	43.33		467.49	301.65	33.61	14.41	16.98	0.17
rats	K24		9.05	32.15	44.42	4.59	944.14	949.55	52.51	16.87	15.86	0.28
rats	K25		7.10	23.13	40.60	2.89	652.71	588.53	49.60	17.54	7.23	0.08
rats	K26	0.06		24.33	42.36	1.94	475.97	513.31	30.74	15.42	55.94	1.19
rats	K27	0.01	9.82	23.39	38.37	11.30	645.15	783.81	48.82	9.18	37.62	0.79
rats	K28					6.19	362.15	291.66	39.67	6.58	58.99	1.53
mice	M1	3.59	4.77	44.52	24.39	41966.59	3752.50		237.81	77.88	53.22	0.32
mice	M2					5716.48	667.05	185.54	43.38	58.20	68.76	0.61
mice	M3	0.60	3.74	40.83	21.14	36429.84	3315.89	565.75	146.93	15.88	125.06	1.32
mice	M4					17922.01	1863.16	464.65	49.72	61.15	45.33	0.64
mice	M5	1.90				18813.55	1458.07	325.56	47.42	23.62	70.41	1.49
mice	M6									44.12	75.01	0.43
mice	M7	2.35	3.61	53.03	28.47	276.09	993.74	158.22	47.79	58.59	136.21	2.32
mice	M8	0.56	3.12	32.25	20.16	345.30	1788.76	418.80	36.04	40.50	186.32	1.05
guinea pigs	GP1	0.01	3.52	34.62	19.28	86.58	1277.08	47.51	1.29	10.50	164.43	0.57
guinea pigs	GP2	0.07	12.39	70.11	20.78	69.83	2014.54	153.61	2.28		89.76	0.38
guinea pigs	GP3		2.95	42.36	22.82	27.84	378.77	75.83	1.57	10.13	181.10	0.81
guinea pigs	GP4	0.04	2.73	60.08	36.64	49.41	595.52	84.86	2.31	0.33	38.90	0.64
guinea pigs	GP5		2.73	20.30	22.27	38.49	350.72	60.88		<LOQ	14.11	0.60
guinea pigs	GP6	0.01	6.00	29.51	13.45	22.07	855.15	25.92	4.88	21.12	29.38	0.12
guinea pigs	GP7	0.12	1.91	65.09	42.16	4.11	53.70	62.38	1.98	1.75	79.32	
guinea pigs	GP8		13.61	79.64	28.33	16.41	644.10	109.77	0.91	1.80	42.36	0.42

TMAO - lungs [nM/g]	TMA - lungs [nM/g]	choline - lungs [nM/g]	carnitine - lungs [nM/g]	TMAO - heart [nM/g]	TMA - heart [nM/g]	choline - heart [nM/g]	carnitine - heart [nM/g]	TMAO - liver [nM/g]	TMA - liver [nM/g]	choline - liver [nM/g]	carnitine - liver [nM/g]	TMAO - renal cortex [nM/g]
5.40	0.71	956.48	291.10	3.60	0.90	293.68	634.81	11.72	6.95	2122.22	182.15	40.43
6.03	0.56	810.13	266.05	4.28	0.79	252.60	650.86	14.44	8.47	1906.01	140.31	21.15
6.66	0.88	959.33	313.04	4.05	0.71	247.04	649.29	35.45	12.05	1392.22	107.40	43.99
7.35	0.65	874.50	322.94	3.95	0.88	247.35	699.96	30.62	10.10	1880.72	129.60	19.41
4.94	0.94	859.51	290.89	3.49	0.66	244.03	641.96	18.23	7.47	1153.57	136.06	35.37
	0.72	844.85	296.98		1.44	271.82	659.80	56.77	14.95	1441.84	169.34	82.30
10.29	0.81	978.31	327.19	3.69	0.79	211.55	694.66	24.60	13.09	1847.08	169.53	22.54
15.95	0.55	814.65	319.23	5.74	1.66	213.90	633.98	28.89	10.09	1789.94	168.17	52.59
30.61	2.90	1045.79	358.06	56.21	12.64	369.84	499.83	6.41	45.83	1998.71	157.71	153.34
	3.02	1274.26	455.29						49.29	2045.66	117.58	136.65
10.91	1.96	1132.61	406.86	9.95	4.11	378.99	425.15	2.97	19.26	4554.12	182.75	24.28
20.77	2.58	1121.32	370.56	9.33	7.05	531.96	429.28	1.94	38.09	2497.93	188.32	14.23
33.20	3.67	1121.06	393.38	6.00	5.79	488.68	453.18	5.23	52.22	4076.36	119.55	16.85
20.61	2.77	1250.45	398.54	7.86	6.33	403.03	454.72	1.41	20.25	5551.21	150.01	7.03
43.03	2.75	1238.23	424.42	13.35	7.55	409.01	485.39	1.69	32.56	4427.23	176.11	92.43
21.86	2.03	1251.13	409.25	48.03	9.40	470.59	572.97	2.04	20.02	2223.87	139.62	11.61
11.71	1.44	1451.63	292.51	2.99	0.06	163.23	475.71	13.05	9.53	3608.07	67.74	23.46
12.38	6.49	1792.44	197.77	7.01	2.19	684.96	725.72		36.24	6236.83	88.52	
2.24	1.71	1470.40	275.29	1.24	0.03	349.35	863.65	13.98	33.56	4178.37	101.41	16.82
3.77	4.54	1623.64	278.16	2.26	0.74	282.26	655.71	9.81	5.74	2754.07	134.49	17.24
3.81	3.77	1238.37	350.85	1.17	0.33	236.85	645.68	7.64	2.76	1819.78	96.17	16.81
10.21	3.85	1454.75	233.79	6.14	3.15	599.17	870.17	15.88	31.33	1722.28	114.50	32.50
4.54	1.13	1343.67	258.27	3.03	1.24	490.44	770.53	3.37	3.17	1446.04	119.07	4.96
8.46	4.00	1361.29	174.13	5.14	1.59	421.85	633.07	7.82	18.52	1271.45	93.68	12.30

TMA - renal cortex [nM/g]	choline - renal cortex [nM/g]	carnitine - renal cortex [nM/g]	TMAO- renal medulla [nM/g]	TMA - renal medulla [nM/g]	choline - renal medulla [nM/g]	carnitine - renal medulla [nM/g]	FMO1/Gapdh mRNA level (a.u.) -liver	FMO1/Gapdh mRNA level (a.u.) -heart
83.08	3168.91	101.06	121.90	57.42	5188.27	94.21	8.72	0.16
29.98	1262.64	100.32	38.15	43.40	3312.56	90.76	10.73	0.20
50.20	825.75	107.16	72.69	31.77	4923.27	102.90	10.81	0.19
58.86	2278.35	92.60	44.80	47.78	3105.80	88.98	10.51	0.29
26.74	1395.40	92.03	48.88	44.66	3644.24	96.69		0.25
129.32	2605.44	84.27	191.33	54.49	5684.30	101.61	13.30	0.20
49.12	3067.10	89.99	52.35	51.35	4406.09	95.71		
90.81	2313.48	101.54	114.11	68.56	4282.07	91.86		
	6209.65	135.69	170.21	142.32	11545.05	141.53	1.08	1.01
74.24	5512.71	117.33	170.02	74.15	11571.20	128.53	1.40	1.20
41.01	6295.09	144.65	41.52	38.79	14166.08	142.96	1.62	1.09
48.31	6732.59	80.56	10.98	44.47	11478.49	96.23	1.51	
49.35	5519.36	89.39	15.99	52.59	8808.83	95.72		1.24
45.76	5243.82	83.86	5.52	73.97	9292.57	103.77	1.09	1.14
79.27	7118.65	123.67	57.14	67.62	13104.72	131.71		
39.65	5662.78	96.26	12.91	118.83	13709.28	114.00		
7.61	5578.92	134.72	30.91	14.33	12680.31	121.11	0.90	0.49
	6925.84	179.91		19.77	9198.24	120.81	0.55	
7.03	5525.36	177.77	18.15	12.46	10545.04	149.64	0.63	0.49
12.31	5342.99	165.15	18.85	13.80	6974.26	133.92	0.43	0.49
7.04	6403.82	217.07	11.56	12.52	8463.75	182.36	1.19	0.45
6.94	4880.18	124.38	32.71	6.82	9812.95	94.05	0.51	0.73
3.77	5882.63	125.13	2.93	8.89	7816.26	113.96		
8.59	5848.73	116.83	17.31	9.38	10749.45	90.59		

FMO1/Gapdh mRNA level (a.u.) -renal medulla	FMO1/Gapdh mRNA level (a.u.) -renal cortex	FMO1/Gapdh mRNA level (a.u.) -lungs	FMO3/Gapdh mRNA level (a.u.) -liver	FMO3/Gapdh mRNA level (a.u.) -heart	FMO3/Gapdh mRNA level (a.u.) -renal medulla	FMO3/Gapdh mRNA level (a.u.) -renal cortex
3.29	3.90	0.07	29.63	0.28	5.83	2.85
2.52	7.30	0.25	36.98	0.24	3.61	5.20
1.74	0.20	0.24	40.47	0.69	3.54	0.15
5.42	2.77	0.03	39.23	0.60	5.54	4.42
	8.63		94.61	0.18		5.17
4.39	2.90	0.11	60.08	0.65	4.42	5.46

1.70	0.98	1.44	27.26	22.53	37.50	36.73
	0.83	1.18	32.53	25.61	26.43	34.27
1.50	0.68	1.78	43.08	37.63	31.86	31.21
0.88	2.49	1.86	30.67	23.00	28.13	27.45
2.01	4.92	1.39	42.63	30.46	29.12	
2.66	2.05	1.76	23.33	19.34	34.87	32.20

0.25	1.18	0.51	2.79	1.96	3.94	1.23
0.83	0.37	0.38	3.81	1.52	2.57	1.58
0.74	0.47	0.41	2.32	1.61	2.98	2.41
0.14	1.03	0.51		4.51	2.39	4.18
0.26	0.43	0.35	2.49	2.31	0.98	2.55
0.43	0.43	0.69	3.15	2.93	4.33	2.73

FMO3/Gapdh mRNA level (a.u.) -lungs	FMO5/Gapdh mRNA level (a.u.) -liver	FMO5/Gapdh mRNA level (a.u.) -heart	FMO5/Gapdh mRNA level (a.u.) -renal medulla	FMO5/Gapdh mRNA level (a.u.) -renal cortex	FMO5 /Gapdh mRNA level (a.u.)-lungs	Relative FMO3 protein level (a.u.) - liver	Relative FMO3 protein level (a.u.) -lungs
0.06	6.32	0.02		0.37	0.03	1.22	0.41
0.39	38.42	0.06	0.94	0.68	0.07	1.34	0.55
0.70	31.43	0.19	1.14	0.04	0.36	1.27	0.47
0.07	37.76	0.11	0.85	0.40	0.03	1.15	0.39
1.06	15.12	0.04		0.93	0.32	1.12	0.49
0.29	17.68	0.09	1.16	0.27	0.09	1.20	0.43
22.30	224.23	53.77	291.80	70.71	75.79	0.37	0.48
37.37	127.46	76.58	409.82	69.98	128.34		0.53
26.89	259.37	112.90	119.33	110.96	140.93	0.37	0.37
40.75	791.73	96.93	68.07	189.87	106.81		0.37
21.61		100.70	239.50		82.65	0.36	0.44
49.48	1037.47	69.50	209.94	107.55	131.95	0.37	0.44
4.89	0.74	0.38	0.56	0.70	1.35	0.92	0.59
1.61	0.99	0.65	0.29	0.30	0.79	1.02	0.45
1.23	0.84	0.39	0.51	1.03	0.84	1.12	0.53
1.90	0.67	1.17	0.37	1.20	0.46	1.15	0.44
2.14	0.96	0.69	0.55	0.69	0.55	1.07	0.42
5.02	0.78	0.74		1.03	1.24	1.04	0.39

Relative FMO3 protein level (a.u.) - renal cortex	Relative FMO3 protein level (a.u.) - renal medulla	Relative FMO5 protein level (a.u.) - liver
1.02	0.95	0.89
0.91	1.00	0.93
0.96	0.95	1.03
1.02	0.99	1.03
0.93	0.95	1.04
0.86		1.09
0.50	0.48	1.26
0.66	0.67	1.25
0.64	0.62	1.33
0.72	0.56	1.33
0.65	0.65	1.25
0.58	0.65	
0.97	1.00	0.19
1.02	0.99	0.20
1.12	1.03	
1.14	1.01	0.21
1.22	1.01	0.18
1.06		0.20
