# Supplementary material for: Mice, rats, and guinea pigs differ in FMOs expression and tissue concentration of TMAO, a gut bacteria-derived biomarker of cardiovascular and metabolic diseases
Source: PLoS One. 2024 Jan 24;19(1):e0297474. doi: 10.1371/journal.pone.0297474 (PMC10807837; doi:10.1371/journal.pone.0297474)

S1 Fig. Western blotting– original images.

Liver – FMO3 and FMO5

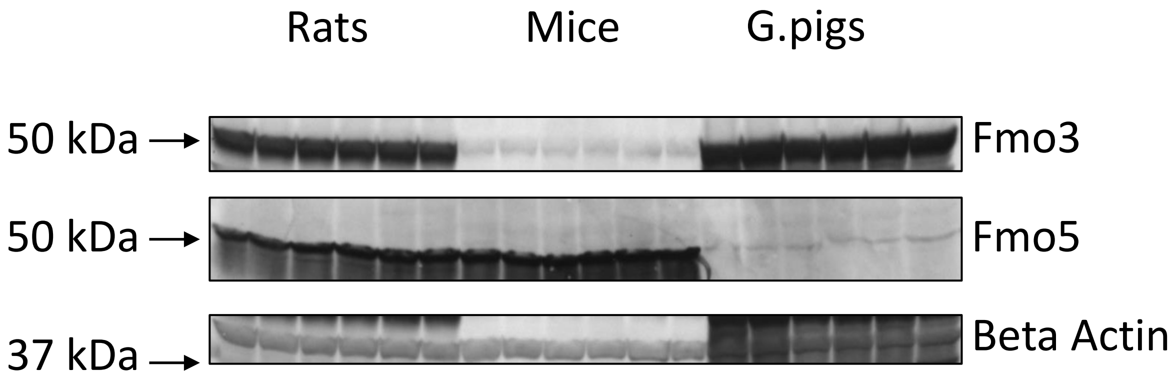

FMO3

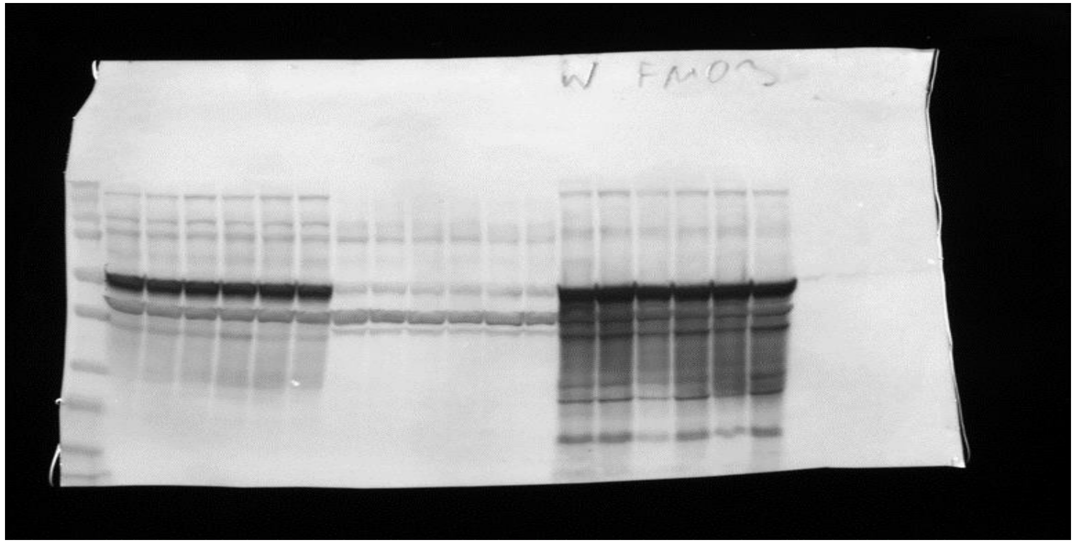

FMO5

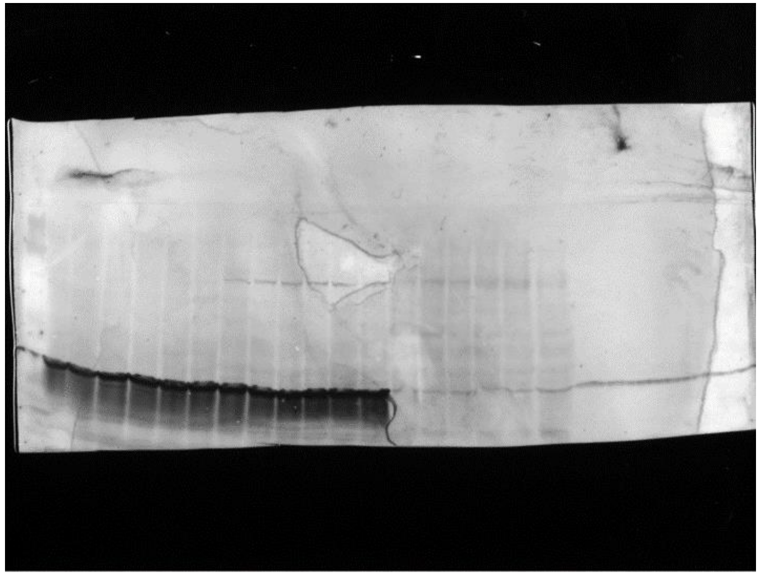

Lungs – FMO3

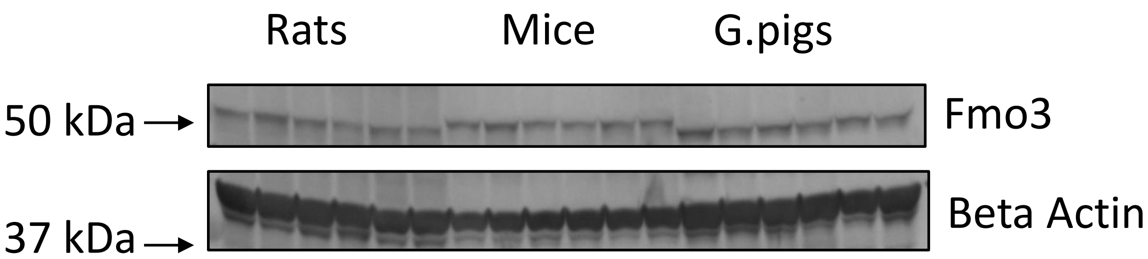

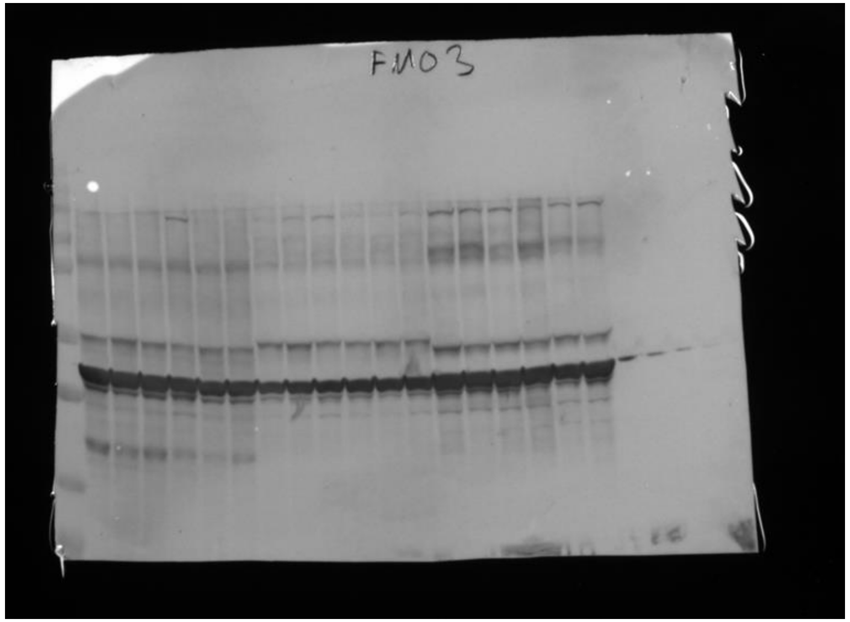

Renal cortex – FMO3

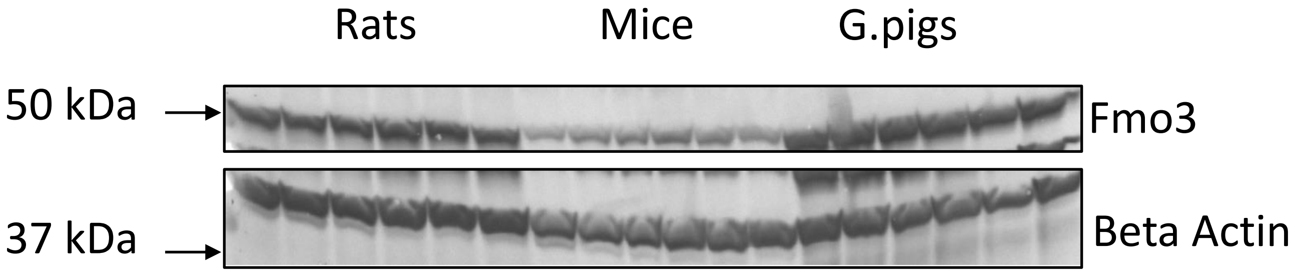

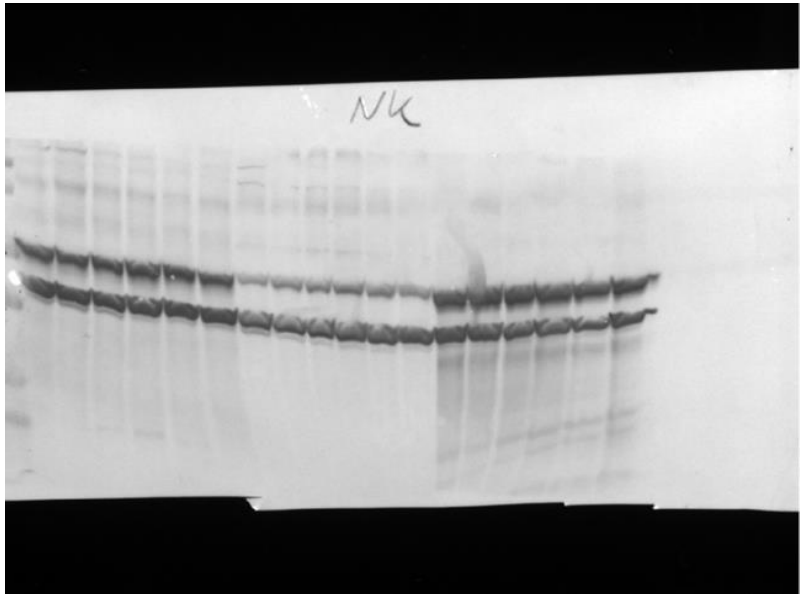

Renal medulla – FMO3

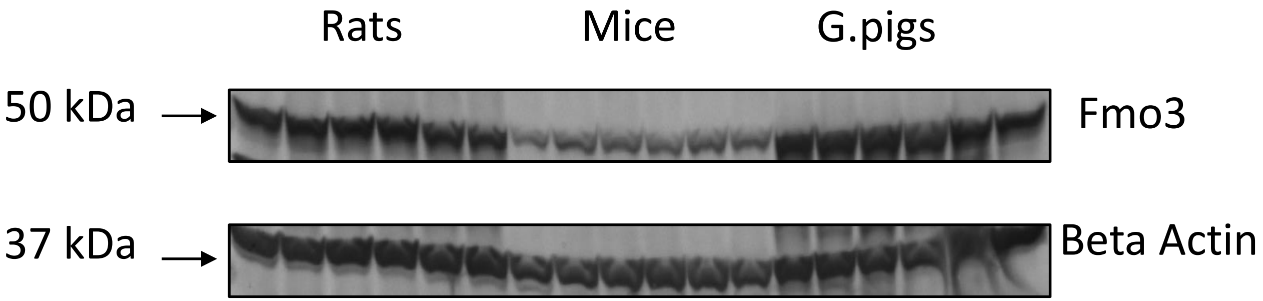

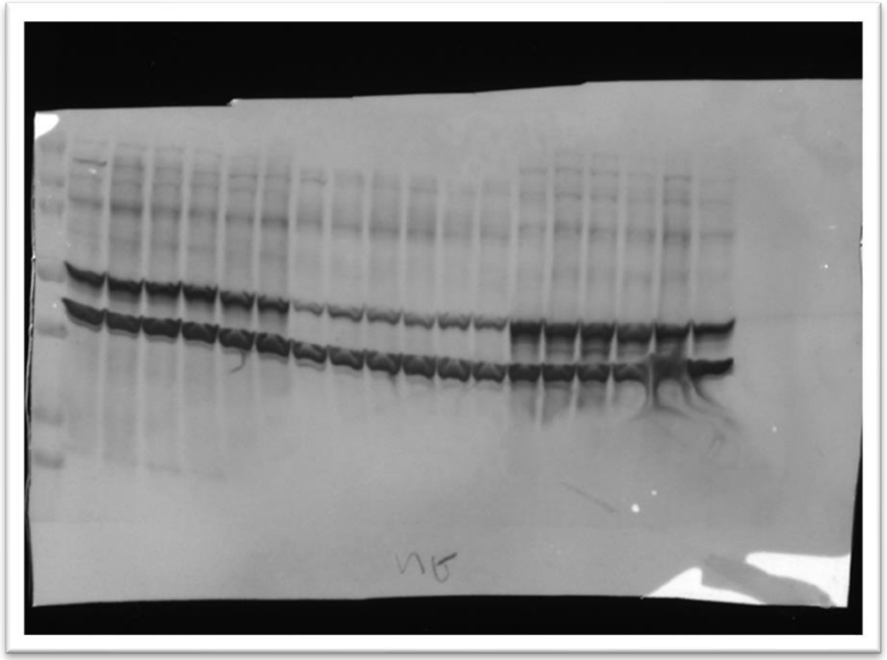

Supplement: S1 Fig — (PDF) [file pone.0297474.s004.pdf]
